# Keystone Taxa and Predictive Functional Analysis of *Sphagnum palustre* Tank Microbiomes in Erxianyan Peatland, Central China

**DOI:** 10.3390/biology11101436

**Published:** 2022-09-30

**Authors:** Baiying Man, Xing Xiang, Junzhong Zhang, Gang Cheng, Chao Zhang, Yang Luo, Yangmin Qin

**Affiliations:** 1 College of Life Science, Shangrao Normal University, Shangrao 334001, China; 2 Key Laboratory of Forest Disaster Warning and Control in Yunnan Higher Education Institutions, South West Forestry University, Kunming 650224, China; 3 State Key Laboratory of Biogeology and Environmental Geology, China University of Geosciences, Wuhan 430074, China

**Keywords:** Erxianyan peatland, *Sphagnum palustre*, core microbiome, function prediction

## Abstract

**Simple Summary:**

Deciphering the relationship between microbiome of keystone species *Sphagnum palustre* and potential function in the Erxianyan peatland ecosystems is important in the context of global peatland degradation. We evaluated the *S. palustre* tank microbiome and predicted the potential ecological functions. In total, 38 phyla, 55 classes, 122 orders and 490 genera were detected. Proteobacteria and Firmicutes are the dominant endophytes in *S. palustre*. Core microbiomes are mainly found in 7 phyla, 9 classes, 15 orders, 22 families and 42 genera. Functions predictive of microbial communities are involved in nitrogen fixation, carbon cycle, nitrate metabolism, sulfate respiration and chitinolysis, which may enable the *Sphagnum* to adapt to harsh environmental conditions. This study provides new insights into the relationship between *Sphagnum*-associated microbiomes and their potential ecological functions in subalpine peatlands.

**Abstract:**

*Sphagnum* is a fundamental ecosystem of engineers, including more than 300 species around the world. These species host diverse microbes, either endosymbiotic or ectosymbiotic, and are key to carbon sequestration in peatland ecosystems. However, the linkages between different types of *Sphagnum* and the diversity and ecological functions of *Sphagnum*-associated microbiomes are poorly known, and so are their joint responses to ecological functions. Here, we systematically investigated endophytes in *Sphagnum palustre* via next-generation sequencing (NGS) techniques in the Erxianyan peatland, central China. The total bacterial microbiome was classified into 38 phyla and 55 classes, 122 orders and 490 genera. The top 8 phyla of Proteobacteria (33.69%), Firmicutes (11.94%), Bacteroidetes (9.42%), Actinobacteria (6.53%), Planctomycetes (6.37%), Gemmatimonadetes (3.05%), Acidobacteria (5.59%) and Cyanobacteria (1.71%) occupied 78.31% of total OTUs. The core microbiome of *S. palustre* was mainly distributed mainly in 7 phyla, 9 classes, 15 orders, 22 families and 43 known genera. There were many differences in core microbiomes compared to those in the common higher plants. We further demonstrate that the abundant functional groups have a substantial potential for nitrogen fixation, carbon cycle, nitrate metabolism, sulfate respiration and chitinolysis. These results indicate that potential ecological function of *Sphagnum palustre* in peatlands is partially rooted in its microbiomes, and that incorporating into functional groups of *Sphagnum*-associated microbiomes can promote mechanistic understanding of *Sphagnum* ecology in subalpine peatlands.

## 1. Introduction

Peatlands play a crucial role in the global carbon cycle. *Sphagnum mosses* are considered to be engineers and contribute to the carbon sequestration in acidic, cold and water-saturated peatland ecosystems [1,2]. The genus *Sphagnum* includes 250–450 species around the world, covering about 4 × 10^6^ km^2^ of peatlands, making it the largest terrestrial carbon reservoir [3,4,5]. As a fundamental member of peatland ecosystems, *Sphagnum mosses* hosts diverse bacterial communities, either endosymbiotic or ectosymbiotic, and therefore, studying them should yield insights into *Sphagnum* biology and the function performed by microbials in peatland ecosystems [1]. Within those *Sphagnum-*associated bacterial communities, some functional groups have begun to be revealed in N_2_ fixation, disease suppression, promote growth [6,7,8] of *Sphagnum* mosses and elemental cycling [9,10] in peatland ecosystems. In addition, the *Sphagnum* microbial biomass and microbial community are impacted by elevated temperatures and respond rapidly to temperature alterations [8,11,12]. Generally, *Sphagnum*-associated microbiomes differ from those of common plants due to the inhospitable acidic environments. Many microbes living in plants can endow hosts with the ability to adapt to extreme conditions, degrade organic pollutants [13], and improve plant growth [14], but little is known about the endosymbiotic nature of *Sphagnum* individuals [15,16,17]. Endophytes are microorganisms that inhabit the internal tissues of plants and have potentially biofunctional interactions with plants. Although *sphagnum* endophytes have been reported continuously in recent years [18,19], they are still relatively unknown compared with the more than 300 other species around the world. 

With the increase in sequencing depth afforded by next-generation sequencing (NGS), studies began focusing on identifying the “core microbiome”. The term “core microbiome” is used to describe the shared microbes common among the microbial communities [20,21,22]. Some core microbiomes are not only common to plant species or habitats [23], but also as important components to perform basic functions [24]. Although studies on core microbiomes are focusing on the key species within human, plant, lake, soil, and wastewater treatment systems [25,26,27,28], there is still a need to bridge the gap between *Sphagnum*-associated microbiomes and *Sphagnum* individuals in parallel with next-generation sequencing (NGS) [29]. 

Erxianyan, a typical subalpine peatland, is located in western China. So far, *Sphagnum-palustre*-associated microorganisms in Erxianyan have been demonstrated only for the diversity and ecology of testate amoebas [30]. In contrast, the endophytes of *S. palustre* in the Erxianyan peatland have yet to be characterized. This substantially limits knowledge acquisition of *Sphagnum*-associated microbiomes in situ as well as further exploration of the potential ecological functions. Thus, the *Sphagnum*-associated microbiomes and their function still remain to be elucidated. Therefore, the objectives of this work were to (1) investigate the diversity and ecology of *S.-palustre*-associated microbiomes in the Erxianyan peatland, central China, (2) determine the *S.-palustre*-associated core microbiomes, and (3) predict its potential ecological functions in subalpine peatlands.

## 2. Materials and Methods

### 2.1. Study Site Description and Sphagnum Collection

**Figure 1:**
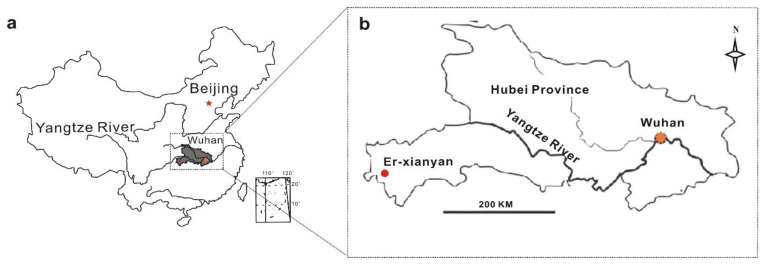
Location of study area (**a**) and sampling site (**b**) in the Erxianyan peatland, central China.

Erxianyan, a subalpine peatland, is located in the Middle Yangtze Reach, western Hubei Province, China (Figure 1a,b). This region is strongly influenced by the East Asian summer monsoon [30,31]. The Erxianyan peatland is considered to comprise ombrotrophic bogs, which receive most of their nutrients and water from precipitation. *Sphagnum palustre* is the dominant vegetation in the Erxianyan peatland based on field investigation combined with laboratory evaluation [32], while other plants include *Malus hupehensis* (Pamp.) Rehd, *Calamagrostis epigeios* (Linn.) Roth, *Carex* sp., *Echinochloa crusgalli* (L.) Beauv, *Hosta ventricosa* (Salisb) Stearm and *Reynoutria japonica* Houtt [30]. 

The *S. palustre* was collected aseptically at five sampling sites (200 m intervals). We collected 6 mixed samples (100 m apart) at each sampling site with sterile tubes (Corning) using the five-point sampling method. Altogether, 30 samples from five sites were collected and named as group A (E0W), group B (E2W), group C (E4W), group D (E10W) and group E (E18W). Temperature (22.1–28.4 °C), water pH (5.3–6.6), dissolved oxygen (0.22 to 0.45 mg/L), electric conductivity (13.2 to 44.1 μS/cm) and oxidation reduction potential (103.7 to 152.2 mV) were measured directly in the field using a multi-parameter water quality detector (HACH, Loveland, CO, USA). When the pore water was still, water table level (−11 to 0 cm) was measured. After being transported to the microbiology lab on ice within 24 h, the matrix was first cleaned by rinsing several times in sterile distilled water to remove the matrix and then washed 3 times for 5 min each time with sterile distilled water. Subsequently, samples were placed into 75% ethanol for 3 min, followed by washing 5 times with sterile distilled water. To ensure that the downstream experiments were all endophytes, the final sterile water was inoculated on the R2A to determine whether disinfection was complete. The residual water was finally absorbed by the sterilized filter paper, and the treated samples were stored at −80 °C in sterile plastic centrifuge tubes (Corning) until DNA extraction.

### 2.2. Genomic DNA Extraction and High-Throughput Sequencing

Total nucleic acids were extracted from 1 g of freeze-dried mixed samples using the PowerSoil DNA Kit (MoBio Laboratories, Inc., Carlsbad, CA, USA). Nucleic acids were eluted with 60 µL buffer and quantified with Nanodrop 2000 (Thermo Fisher Scientific, Waltham, MA, USA). Diluted DNA (1 ng/µL) was used as PCR templates and RNAase-free water as negative controls. To access the bacterial communities, three sets of index primers were used to amplify the V4 (515F–806R) region of the 16S rRNA gene of each sample [33]. PCR reactions contained a total volume of 50 μL, including 25 μL Premix Taq (Takara Biotechnology, Dalian Co., Ltd., Dalian, China), 1 μL of each primer (10 mM) and 3 μL DNA (20 ng/μL) template and 20 μL RNAase-free water. The PCR conditions were as follows: 95 °C for 3 min and 27 cycles of 95 °C for 30 s, 55 °C for 30 s and 72 °C for 45 s, with a final extension of 72 °C for 10 min. Products were purified and quantified with a Quit 2.0 fluorometer (Invitrogen, Carlsbad, CA, USA). Libraries of samples were generated using Ion Plus Fragment Library Kit 48 rxns (Thermo Fisher Scientific, Shanghai Co., Ltd., Shanghai, China) and assessed on the Qubit@ 2.0 Fluorometer (Invitrogen, Carlsbad, CA, USA) and Agilent Bioanalyzer 2100 system. Sequencing was then performed commercially on the IonS5TMXL sequencing platform at the Novogene Bioinformatics Technology (Beijing, China).

### 2.3. Bioinformatics Analysis

Briefly, the low-quality parts of reads were discarded by Cutadapt (V1.9.1, http://cutadapt.readthedocs.io/en/stable/, accessed on 5 March 2022) [34]. Raw reads were quality-filtered and de-multiplexed. Chimeric sequences were removed by VSEARCH [35]. Operational taxonomic units (OTUs) were assigned using UPARSE (version 7.1 http://drive5.com/uparse/, accessed on 5 March 2022) with a 97% cut-off [36]. The high-quality sequences were aligned against the SILVA database (http://www.arb-silva.de/, accessed on 13 September 2021) [36,37] for taxonomic classification. Diversity indices, such as Observed-species, Chao1, ACE, Shannon, Simpson and Good’s coverage, were calculated with rarefied data using QIIME (Version 1.7.0). Species accumulation curves were created using R software. Differences in alpha diversity for all pairwise differences between means were compared via Tukey’s and Wilcox tests. Dissimilarity comparisons in each two sampling sites were further examined under significant values (α = 0.05) using PERMANOVA based on Bray-Curtis dissimilarities, and the *p*-value was adjusted via the Benjamini method. Differences in alpha diversity were tested with one-way analysis of variance (ANOVA) in SPSS 18. Community composition was performed via PCoA (principal co-ordinates analysis) in R software (Version 2.15.3). The distinctiveness of microbiomes in different sites was analyzed using the linear discriminant analysis effect size (LEfSe) method. The taxon with significant differences between groups and significance of the detected variations were analyzed via *t*-test (*p*-value).

The composition of the core microbiome (the OTUs observed in most (≥90%) of the samples) was calculated to present the diversity of the bacterial community at a more refined taxonomic level. We submitted the OTU biome file to MetaCoMET (the Metagenomics Core Microbiome Exploration Tool) for discovery and visualization of the core microbiomes [38]. A significance test was performed using ANOVA, and a *p* value < 0.05 was considered statistically significant. In addition, FAPROTAX was constructed for functional annotation of microbiomes. 

All the sequencing data were deposited in the National Omics Data Encyclopedia (NODE) under the project accession OEP001043 (https://www.biosino.org/node/project/detail/OEP001043, submitted on 3 July 2020).

## 3. Results

### 3.1. Community Composition and Biodiversity Assessment

**Figure 2:**
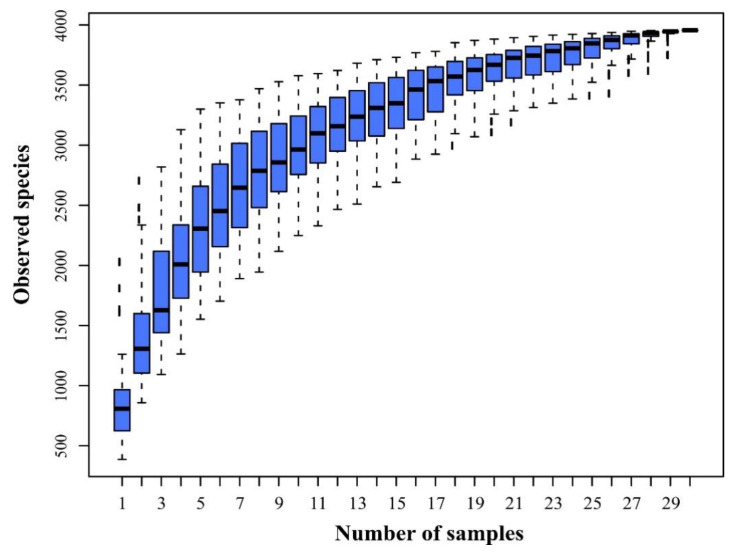
Species accumulation boxplot for sequencing samples of *S. palustre* in the Erxianyan peatland.

A total of 30 *S. palustre* samples in the Erxianyan peatland were sequenced using the IonS5^TM^XL platform, resulting in 984 Mb reads and 2,186,399 sequences. Of those, 2,110,195 (96%) high-quality chimera-free clean data passed the stringent quality control (Phred quality scores 20: 96.5% on average) and fell into 1,851,366 taxa tags (61,712 on average) and 3969 OTUs (Table 1). The mean OTUs ranged from 499 to 2152, with a 3% cutoff. A total of 3969 OTUs were annotated based on ≥97% nucleotide sequence identity between sequences, including 38 phyla, 55 classes, 122 orders and 490 genera. Only 0.05% of sequences belong to an unclassified group. Species accumulation curves were generated for each sample to assess whether the sample size provided sufficient OTU coverage of the *Sphagnum*-associated microbiomes (Figure 2). The species accumulation curves showed that the libraries could reflect the main bacterial information in each sample (Figure 2). 

**Figure 3:**
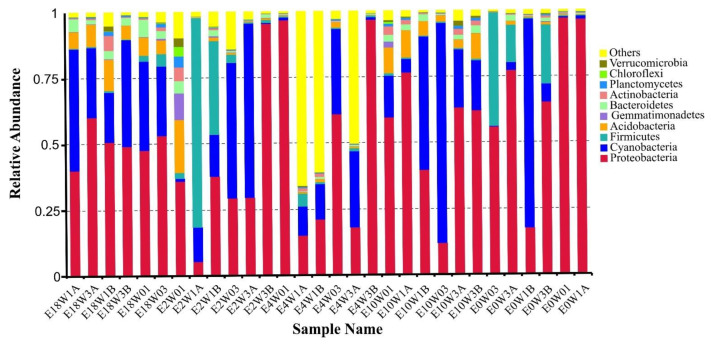
The relative abundance of top 10 phyla of endophytes detected from *S. palustre* in the Erxianyan peatland.

Of 38 detected phyla, Acidobacteria, Actinobacteria, Bacteroidetes, Cyanobacteria, Firmicutes, Proteobacteria and Gemmatimonadetes were distributed as common phyla in all samples. Further analysis indicated that Proteobacteria was the dominant phylum, accounting for 48.26 and 33.69% of total reads and OTUs. It was subdivided into three classes: gamma, alpha and delta. The top 8 phyla (the relative abundance > 1%) occupied 78.31% of the total OTUs, including Proteobacteria (33.69%, 1337 OTUs), Firmicutes (11.94%, 474 OTUs), Bacteroidetes (9.42%, 374 OTUs), Actinobacteria (6.53%, 259 OTUs), Planctomycetes (6.37%, 253 OTUs), Gemmatimonadetes (3.05%, 121 OTUs), Acidobacteria (5.59%, 222 OTUs) and Cyanobacteria (1.71%, 68 OTUs) (Figure 3). The remaining 30 bacterial taxa were rare and only accounted for 1.28% and 21.69% of the relative abundance and OTUs, respectively.

At the order level, a total of 122 orders were obtained from the Erxianyan peatland. The top 12 orders (the relative abundance > 1%) include 9 known orders and 3 unidentified groups. Of those, the relative abundance of unidentified_Cyanobacteria (27.16%) was the dominant group, followed by unidentified gamma Proteobacteria (18.19%), Xanthomonadales (12.71%), unidentified alpha Proteobacteria (12.17%), Lactobacillales (4.43%), Clostridiales (3.34%), Caulobacterales (3.00%), Acidobacteriales (2.85%), Rhizobiales (2.51%), Rickettsiales (2.24%), Sphingomonadales (1.66%) and Enterobacteriales (1.10%). The top 12 orders covered nearly 77.78% and 31.49% of total reads and OTUs, respectively. The remaining 106 bacterial taxa were rare (the relative abundance < 1%), and the relative abundance only accounted for 8.60%.

**Figure 4:**
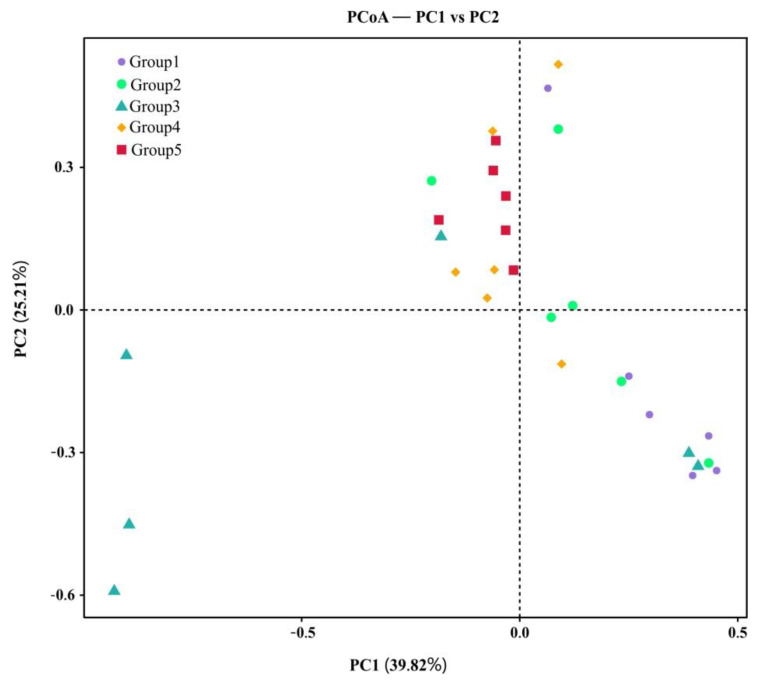
PCoA (weighted−UniFrac distance) of *S. palustre* endophytes in the Erxianyan peatland.

Microbiomes were highly diverse in *S. palustre*, as indicated by alpha diversity. The dominant phylotypes were fully captured by high-throughput sequencing, as evidenced by a high Good’s coverage (from 99.40% to 99.70%, 99.62% on average, Table 1) (n = 30) and plateaued species accumulation curves (Figure 2). No significant difference was observed for the Chao1 (503.61 to 2092.96, Mean = 1056.75) and ACE (549.77 to 2108.92, Mean = 1091.62) indices. Shannon’s index ranged from 2.40 to 9.19 (4.77 on average) among 30 samples (Table 1). Community diversity had a significant difference across sampling sites, especially in Shannon’s index for the A and D (Wilcox test, *p* = 0.0049), A and E (Wilcox test, *p* = 0.0027) and C and E groups (Wilcox test, *p =* 0.0043). The Simpson index, however, had a significant difference only in the A and D (Wilcox test, *p* = 0.0206) and A and E groups (Wilcox test, *p* = 0.0183). Alpha diversity had a significant difference between the B and E groups (ANOVA, *p* = 0.004). The microbiome between two sites had a significant difference, e.g., the A and E (PERMANOVA, *p* = 0.03, *F*_value_ = 5.77) groups and C and E (PERMANOVA, *p* = 0.03, *F*_value_ = 13.49) groups. PCoA based on the weighted-UniFrac distance explained 39.82% of the variation through Axis 1 and 25.21% through Axis 2 (Figure 4).

The application of LEfSe analysis can help to find indicator groups in various sites. In total, 30 indicator groups were distinguished from the 5 sampling sites and were mainly associated with four classes of gamma Protobacteria, alpha Protobacteria, Bacteroidia and Acidobacteriia. Group E identified 15 indicator groups, such as genera of *Acidisoma, Mucllaglnibacter, Bacteroidia, Granulicella, Rhodanobacter, Acidocella* and the family of Acetobacteraceae and Sphingobacteriaceae. Conversely, only seven indicator groups were detected in group B, including Bacilli, Lactobacillales, Enterobacteriales Enterobacteriaceae, Lactobacillaceae, Lactobacillus and Cupriavidus. Gamma Protobacteria (*t*-test, *p* = 0.012) and alpha Protobacteria (*t*-test, *p* = 0.004) had a relatively high abundance in group E, while Bacteroidia (*t*-test, *p* = 0.003) and Acidobacteriia (*t*-test, *p* = 0.03) had a relatively high abundance in group D.

### 3.2. The S.-palustre-Associated Core Microbiome

Microbiomes of *S. palustre* in the Erxianyan peatland are highly diverse, with 491 genera in total. We further selected core OTUs (OTU shared by most (≥90%) sample individuals) to present the bacterial community diversity at a more refined taxonomic level, such as known genus. In all, 183 core OTUs were selected and comprised up to 71.85% and 4.95% of total reads and OTUs, respectively. We found that the core microbiome was mainly distributed in 7 phyla, 9 classes, 15 orders, 22 families and 42 genera. Of them, 43 core known genera of 183 core OTUs were examined (Online Resource, Table S1). These core known genera, which in total accounted for 0.01–8.53% of total reads of core OTUs, showed clear dominant core known genera mainly in three phyla: Proteobacteria (24.98%, 50 OTUs), Firmicutes (4.54%, 11 OTUs) and Acidobacteria (1.59%, 4 OTUs). Proteobacteria was by far the most common and comprised up to 24.98% of the core bacterial microbiome in *S. palustre* at the phylum level (Figure 3). At the genus level (Online Resource, Table S1), the seven high-abundance genera (relative abundance >1%, bold in Online Resource, Table S1) of *Stenotrophomonas* (8.53%), *Dyella* (3.86%)*, Lactobacillus* (3.39%), *Acidocella* (3.18%)*, Acidisoma* (2.18%)*, Granulicella* (1.42%) and *Rhodanobacter* (1.38%) and the six medium-abundance genera (relative abundance > 0.5%, bold in Online Resource, Table S1) of *Lactococcus, Sphingomonas, Acidisphaera, Roseiarcus, Serratia* and *Pseudomonas* encompassed nearly 27.52% and 19.13% of total reads and OTUs of 183 core OTUs, respectively, and relative abundances varied from 0.54% to 8.53%. The remaining genera were rare (relative abundance < 0.5%) and mainly distributed across seven phyla (Online Resource, Table S1).

**Figure 5:**
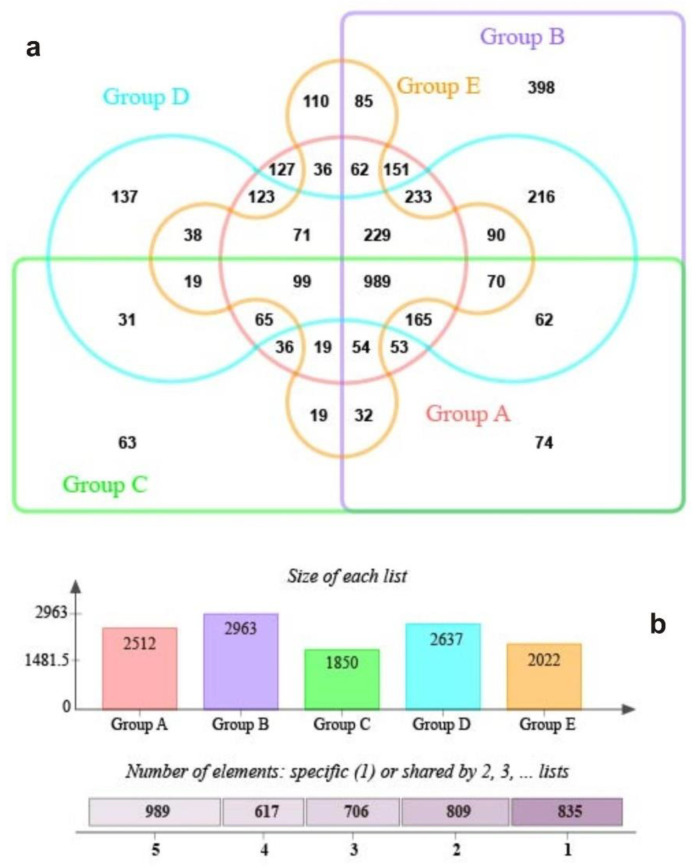
Venn diagram analysis of the total number of common and distinct OTUs (**a**) and sequences (**b**) for *S. palustre* endophytes in the Erxianyan peatland.

**Figure 6:**
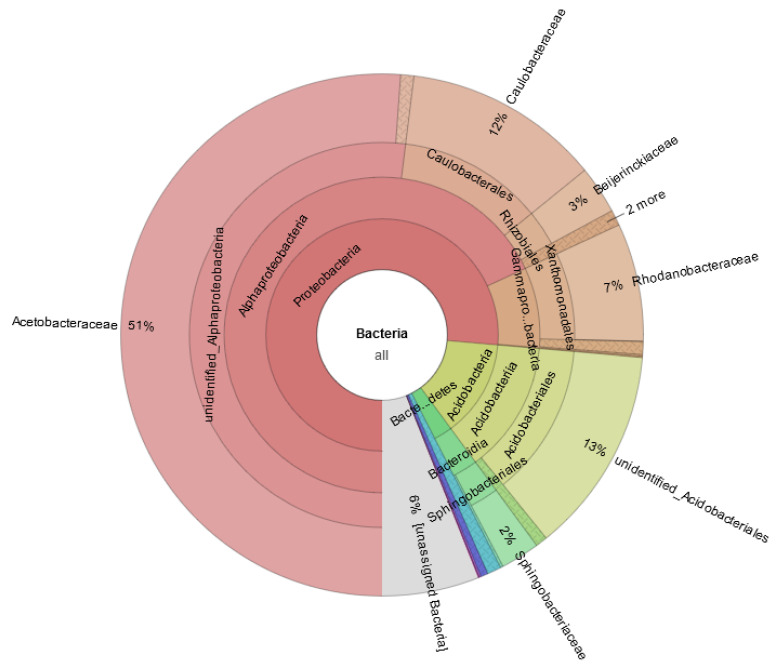
Taxonomic compositions of 75 OTUs at different phylogenetic levels using MetaCoMET. Circles go from inside to outside, indicating different taxa (kingdom to genus). The percentage represents the relative abundance of OTUs.

In addition, we used the persistence method to identify the OTUs present in 30 samples and determine the core microbiome of *S. palustre* on the MetaCoMET platform. In all, 989 shared OTUs and unique OTUs for five sample groups were statistically shown in the Venn graph (Figure 5a). Unique or shared OTUs for each group are displayed in Figure 5b. Of them, 75 OTUs exhibited statistically significant differences between OTU abundance in different sample groups (ANOVA, *p* < 0.05); the hierarchical taxa are displayed in Figure 6. Of them, 13 known genera, i.e., *Acidipila*, *Acidisoma*, *Acidisphaera*, *Acidocella*, *Conexibacter*, *Granulicella*, *Methylocella*, *Mucilaginibacter*, *Novosphingobium*, *Phenylobacterium*, *Rhodanobacter*, *Roseiarcus* and *Singulisphaera*, showed statistically significant differences in different sample groups (ANOVA, *p* < 0.05), many of which were consistent with the known genus of 90% of the samples (Figure 6, Online Resource, Table S1). Notably, we found that 10 genera of *Acidicapsa*, *Alistipes*, *Aquisphaera*, *Arenimonas*, *Bryobacter*, *Gemmata*, *Inquilinus*, *Ralstonia*, *Rhodovastum* and *Terriglobus* still showed statistically significant differences for groups (ANOVA, *p* < 0.05), but they were not present in each individual sample.

### 3.3. Functional Prediction of Microbial Communities

**Figure 7:**
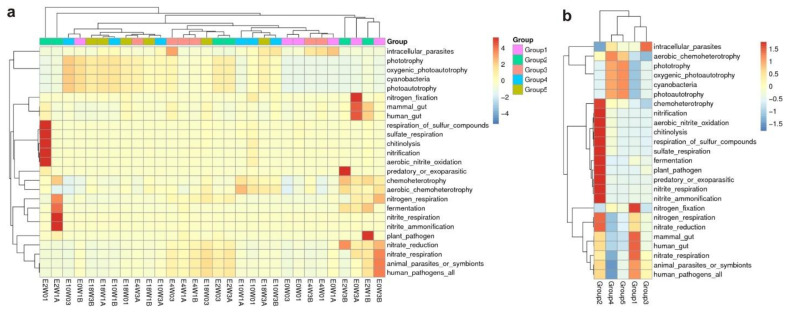
Clustering of functional prediction based on samples (**a**) and sample groups (**b**) for microbiomes of *S. palustre* based on the FAPROTAX database.

Functional annotation of *S.-palustre*-associated microbiomes revealed a rich repertoire of ecological function groups. A hierarchically clustered heatmap of the top 25 bacterial ecological functional groups was established (Figure 7a,b). The refined taxa of microbial groups and their potential functions are shown in an online resource, Table S2. Phototrophy, photoautotrophy, nitrogen fixation, nitrogen respiration, nitrification, nitrite respiration, nitrite ammonification, aerobic nitrite oxidation, nitrate reduction, nitrate respiration, fermentation, aerobic chemoheterotrophy, chemoheterotrophy, sulfate respiration, respiration of sulfur compounds and chitinolysis were generally abundant, indicating that both heterotrophs and autotrophs are important members of the *S. palustre* microbial communities (Figure 7a,b). We note that the predominant ecological function groups were aerobic chemoautotrophs and chemoheterotrophs, indicating that the oxidation of organic compounds is the main source of carbon and energy. In our study, we detected 13 ecological function groups related to the C cycles, including chemoheterotrophy, aerobic chemoheterotrophy, phototrophy, photoautotrophy, photoheterotrophy, cellulolysis, aromatic compound degradation, oxygenic photoautotrophy, methylotrophy, methanotrophy, methanol oxidation, hydrocarbon degradation and aromatic hydrocarbon degradation (Online Resource, Table S2).

Moreover, we note that the microbiomes of some samples were potentially involved in aerobic ammonia oxidation, denitrification and respiration of nitrogen compounds, including all nitrogen cycling steps, such as nitrification, denitrification, aerobic ammonia oxidation, nitrate reduction, nitrate respiration, nitrogen fixation, nitrogen respiration, aerobic nitrite oxidation, nitrite respiration, nitrous oxide denitrification, nitrate denitrification and nitrite denitrification (Figure 7a,b; Online Resource, Table S2). We also detected the functional group of nitrate denitrification and nitrite denitrification in 93% of the samples, which is the main step of the nitrogen loss pathway. Further, microbial communities in 93% of the samples also contained ureolysis, an ammonia-producing precursor to nitrification. 

## 4. Discussion

### 4.1. Proteobacteria Dominant in S.-palustre-Associated Microbiomes

Bacterial communities play an important role in peatland ecosystems, and therefore, studying their dominant phyla should yield an in-depth understanding of their key roles. In our study, 491 genera, 122 orders and 55 classes were detected in 38 phyla. Of them, Proteobacteria dominated in *S*.-*palustre*-associated microbiomes, which corroborates prior works on the *Sphagnum* in other peatlands [18,39,40]. Recently, studies have implicated Proteobacteria populations as the dominant microbial taxa in peatlands [41] and found that they are inextricably linked to higher carbon availability in acidic environments [42]. Some of them have growth-promoting functions for *Sphagnum* and deeply affected the ecological function of peatland ecosystems [10]. The high abundance of Proteobacteria in *S.-palustre*-associated microorganisms could play a key role in the carbon cycle in the Erxianyan peatland.

In our survey, only three classes of Proteobacteria were detected in all samples, namely alpha, gamma and delta Proteobacteria, which accounted for 11.41%, 13.73% and 7.23% of total OTUs, respectively. *Sphagnum-palustre*-associated gamma Proteobacteria was dominant in Erxianyan. This result represents a significant difference from previous reports [19,43], which showed a predominance of alpha Proteobacteria in the Dajiuhu peatland. It has been demonstrated that gammaProteobacteria is ecologically diverse and richer in genera than all bacterial phyla except Firmicutes. This result is further echoed by our dataset of 205 known genera which belong to the class of gamma Proteobacteria, except for the unclassified groups. To note, the high relative abundance of alpha and gamma Proteobacteria in Erxianyan is consistent with that in tropical peatlands [44]. This result indicates that these populations may be specialized for subtropical and tropical peatlands. 

### 4.2. The S.-palustre-Associated Core Microbiome

The core microbiome varies greatly in different niches but generally maintains the biodiversity and stability of ecosystems [22,27,45,46]. It has been demonstrated that the core microbiome does not only enhance resistance against environmental stress [47] and produce flavors for vinegar [38,48] but also produces a secondary metabolite [27,28]. In this study, the core microbiome was detected in more than 90% of *S. palustre* samples, including 43 known genera in 7 phyla. However, of the known genera in our dataset, Pseudomonadales mainly comprise *Roseiarcus*, *Acidocella*, *Serratia*, *Pseudomonas* and *Dyella*, and they seem to play a potential role in the degradation of aromatic compounds. As indicator groups for *S. palustre*, *Serratia* and *Pseudomona* were also detected in the Dajiuhu peatland [49]. Moreover, *Stenotrophomonas*, which had the highest relative abundance within the known genera, is prone to reduction of nitrate. 

In addition to those listed above, 30 more known genera were shared by more than 90% of the samples, with a relative abundance that ranged from 0.01% to 0.45%. *Singulisphaera* is an aquatic bacterium that often inhabits fresh water and acidic *Sphagnum*-dominated wetlands [50], and it is still being examined in this study. *Lactobacillus*, *Lactococcus*, *Enterococcus*, *Bacillus*, *Sporosarcina* and *Butyrivibrio* were found in more than 90% of the samples; however, they are rarely detected in higher plants [51] (Alica et al., 2019). Therefore, there were many differences in the *Sphagnum*-associated core microbiome compared to those in the common higher plants. 

Notably, the phylum Gemmatimonadetes was detected in all samples, although it was not listed in core microbiomes due to the lack of an identified genus at a more refined taxonomic level. Previous studies have also shown that this group is usually found in grassland soils, prairie soils and sediment environments [52,53,54] (Ma et al., 2018; Peng et al., 2017; Zinke et al., 2018). Its long-term evolution has made *S. palustre* an ideal host for Gemmatimonadetes [55]. Therefore, the possible functions of Gemmatimonadetes in *S. palustre* are still not clear and worth further study.

### 4.3. Methodological Limitations and Future Aspects

The *Sphagnum-*associated microbiome and its interactions provide an important reference for understanding the potential ecological functions of microbiomes in peatlands. Nevertheless, our results may be influenced by multiple factors, such as soil pH, organic matter, water table, climate and human practices. To uncover the relationship between microbes and *Sphagnum*, comprehensive environmental factors, more detailed monitoring of various parameters and the accumulation of annual data should be considered in the near future [2,40,49]. However, it should be noted that although the prediction tool FAPROTAX can be used for a fast-functional screening of 16S derived microbiome data from *S. palustre (*Figure 7*),* the actual ecological function in the Erxianyan peatland still needs to be verified after screening assays. Further isolation and identification of more potentially biofunctional endophytes in *S. palustre* will still be needed [18]. In addition, it is worthwhile to determine the ecological framework of microbiome, mycobiome and archaea in the future. 

## 5. Conclusions

Microbial communities play a crucial role in peatland ecosystems. We detected the *S.-palustre-*associated core microbiomes in the Erxianyan peatland, which were mainly distributed across 7 phyla, 9 classes, 15 orders, 22 families and 42 genera. We analyzed the potential ecological functions that might enable the hosts to adapt to harsh environmental conditions. We found some common or unique taxa of *S.-palustre*-associated microbiomes in different groups. The potentially biofunctional endophytes in *S. palustre* are involved in nitrogen fixation, carbon cycling, nitrate metabolism, sulfate respiration and chitinolysis, which may enable *Sphagnum* to adapt to harsh environmental conditions. Although the predominant phylum is the same as others reported, there are still some subdominant groups that differ from those in other subalpine peatlands. Moreover, some groups were unique to *Sphagnum* individuals compared to those in the common higher plants. Certainly, further studies on the interaction mechanism between more *Sphagnum* genotype individuals and microbes are required, and the potential ecological functions in the hosts still need to be determined. 

## Figures and Tables

**Table 1:** The sequencing results of microbiomes in *S. palustre* in the Erxianyan peatland. Diversity indices and richness metrics are also presented.

Sample	Total Tags	Taxon Tags	OTUs	Observed Species	Shannon	Simpson	Chao1	ACE	Goods Coverage (%)
E0W01	70,028	64,973	1015	809	3.34	0.77	994.25	1000.77	99.60
E0W03	70,144	65,305	499	386	2.76	0.77	503.61	549.77	99.70
E0W1A	70,260	64,355	753	615	3.93	0.82	726.78	769.59	99.70
E0W3A	70,137	65,126	735	597	4.91	0.92	697.72	717.74	99.70
E0W1B	75,644	71,054	926	770	2.78	0.57	917.00	998.60	99.60
E0W3B	70,168	65,832	1466	1261	4.16	0.76	1415.29	1460.88	99.50
E2W01	70,086	58,693	2152	2028	9.19	1.00	2092.96	2108.92	99.70
E2W03	70,098	59,469	1109	914	4.25	0.82	1059.44	1106.76	99.60
E2W1A	70,197	66,422	615	497	2.40	0.57	613.04	653.66	99.70
E2W3A	70,736	66,471	779	624	2.95	0.69	768.01	782.86	99.70
E2W1B	62,775	55,600	1770	1610	5.59	0.91	1644.79	1703.36	99.70
E2W3B	68,365	63,327	748	601	4.42	0.91	722.26	753.98	99.70
E4W01	70,121	65,995	857	681	2.54	0.64	852.01	893.87	99.60
E4W03	70,039	64,692	911	762	5.00	0.91	907.76	955.94	99.60
E4W1A	70,135	30,225	1021	854	4.23	0.81	975.72	1015.33	99.60
E4W3A	75,810	38,562	775	644	4.11	0.86	791.02	831.05	99.70
E4W1B	70,114	32,545	927	773	4.25	0.81	920.86	952.33	99.60
E4W3B	70,141	65,377	714	576	3.22	0.77	728.80	754.16	99.70
E10W01	70,168	63,701	1995	1809	7.77	0.99	1996.37	2004.69	99.40
E10W03	70,113	66,461	1030	852	2.98	0.57	1007.37	1035.15	99.60
E10W1A	70,138	64,424	1057	901	6.40	0.98	1049.68	1081.34	99.60
E10W3A	70,131	64,665	1072	927	6.38	0.96	1046.72	1068.99	99.70
E10W1B	73,679	69,889	851	701	4.55	0.84	805.33	851.42	99.70
E10W3B	70,130	64,983	1136	1004	6.61	0.97	1157.46	1166.40	99.60
E18W01	70,162	65,309	1349	1169	5.74	0.91	1323.50	1367.93	99.50
E18W03	70,195	65,172	1833	1633	6.20	0.92	1804.13	1840.30	99.40
E18W1A	70,053	65,817	940	795	5.13	0.86	952.96	984.06	99.60
E18W3A	70,081	65,481	1114	921	5.31	0.92	1116.03	1167.87	99.50
E18W1B	70,213	64,520	1413	1227	7.00	0.97	1397.65	1435.94	99.50
E18W3B	70,134	66,921	704	602	5.09	0.89	714.13	735.06	99.70
**Total**	2,110,195	1,851,366	32,266	27,543					
**Mean**	70,340	61,712	1076	918	4.77	0.84	1056.75	1091.62	99.62

## Data Availability

The data are available from the National Omics Data Encyclopedia (accession OEP001043, https://www.biosino.org/node/project/detail/OEP001043).

## Supplementary Materials

The following supporting information can be downloaded at: https://www.mdpi.com/article/10.3390/biology11101436/s1, Table S1: The taxonomy information of 42 known genera in core microbiomes which shared with most (≥90%) of the samples in Erxianyan peatland, central China; Table S2: The predominant ecological function of the *Sphagnum palustre* associated microbiomes in Erxianyan peatland, central China.
